# Sex-based impact of pancreatic islet stressors in Glu^CreERT2^/Rosa26-eYFP mice

**DOI:** 10.1530/JOE-23-0174

**Published:** 2023-09-25

**Authors:** Neil Tanday, Aimee Coulter-Parkhill, R Charlotte Moffett, Karthick Suruli, Vaibhav Dubey, Peter R Flatt, Nigel Irwin

**Affiliations:** 1 Diabetes Research Centre, Ulster University, Coleraine, Londonderry, Northern Ireland

**Keywords:** sex, islet, insulin, streptozotocin, hydrocortisone, transdifferentiation

## Abstract

The present study examines differences in metabolic and pancreatic islet adaptative responses following streptozotocin (STZ) and hydrocortisone (HC) administration in male and female transgenic Glu^CreERT2^/Rosa26-eYFP mice. Mice received five daily doses of STZ (50 mg/kg, i.p.) or 10 daily doses of HC (70 mg/kg, i.p.), with parameters assessed on day 11. STZ-induced hyperglycaemia was evident in both sexes, alongside impaired glucose tolerance and reduced insulin concentrations. HC also had similar metabolic effects in male and female mice resulting in classical increases of circulating insulin indicative of insulin resistance. Control male mice had larger pancreatic islets than females and displayed a greater reduction of islet and beta-cell area in response to STZ insult. In addition, female STZ mice had lower levels of beta-cell apoptosis than male counterparts. Following HC administration, female mouse islets contained a greater proportion of alpha cells when compared to males. All HC mice presented with relatively comparable increases in beta- and alpha-cell turnover rates, with female mice being slightly more susceptible to HC-induced beta-cell apoptosis. Interestingly, healthy control female mice had inherently increased alpha-to-beta-cell transdifferentiation rates, which was decreased by HC treatment. The number of glucagon-positive alpha cells altering their lineage to insulin-positive beta cells was increased in male, but not female, STZ mice. Taken together, although there was no obvious sex-specific alteration of metabolic profile in STZ or HC mice, subtle differences in pancreatic islet morphology emphasises the impact of sex hormones on islets and importance of taking care when interpreting observations between males and females.

## Introduction

Epidemiological data suggest that males are at increased risk of developing type 2 diabetes (T2D) than females, but that this sex bias is absent with onset of the menopause (Saeedi *et al.* 2020). Clearly, there is an implication that oestrogen acts as a protective factor for pancreatic islet beta cells to help prevent development of T2D (Tiano & Mauvais-Jarvis 2012). Beyond this, males and females also show divergent responses in terms of diabetes complications, efficacy of pharmacological interventions and drug metabolism (Kautzky-Willer *et al.* 2023). This sexual dimorphism is poorly understood for various reasons. First, preclinical assessment of novel antidiabetic compounds generally tends to be conducted in male animals to circumvent postulated metabolic variabilities associated with the oestrus cycle (Bailey & Matty 1972, Richardson *et al.* 2015). Second, prior to 1993, U.S. Food and Drug Administration guidance for clinical testing of new drug entities recommended initial assessment almost exclusively in male populations (FDA 1993). Such matters were highlighted with devastating effects in the well-publicised thalidomide cases of the 1950s, resulting in the death of 2000 children and severe birth defects in thousands more (Vargesson 2015). Preclinical testing of the drug using female tissue or animals could have predicted the teratogenic nature of thalidomide (Therapontos *et al.* 2009). Despite efforts to promote clinical assessment in wider demographic profiles, women of child-bearing age are still under-represented in early clinical trials (Steinberg *et al.* 2021). As a result, approved drugs can still fail in the real-world setting due to undetected female specific side effects (Zucker & Prendergast 2020). It follows that preclinical trials should be designed to fully account for possible sex-based dimorphic impact, necessitating a greater appreciation of the underlying differences in male and female models of disease.

In this respect, the impact that sex exerts in the response to streptozotocin (STZ) or hydrocortisone (HC) administration, frequently adopted approaches to chemically induce contrasting aetiologies of metabolic dysregulation in rodents, is not fully understood. As such, STZ is a toxic glucose analogue that is selectively taken up by beta cells via the GLUT2 transporter (Lenzen 2008). Within the beta-cell STZ alkylates proteins and fragments DNA culminating in beta-cell death and insulin-dependent diabetes (Lenzen 2008). Interestingly, female mice are considered to be less susceptible to STZ-induced diabetes than male counterparts (Kolb 1987, Kim *et al.* 2020), possibly due to oestrogen mediated beta-cell protective effects. However, others have questioned this opinion and highlight that female rodents are still equally susceptible to STZ-induced diabetes as male mice (Plesner *et al.* 2014). On the other hand, glucocorticoids, such as hydrocortisone (HC) and prednisolone, are recognised to induce insulin resistance and to some extent adversely alter insulin secretion (Hansen *et al.* 2010). In peripheral tissues, prolonged glucocorticoid administration increases hepatic glucose production (Dirlewanger *et al.* 2000) and reduces glucose uptake by decreasing GLUT4 expression (Saad *et al.* 1993). In addition, within pancreatic beta cells, glucocorticoids have been demonstrated to inhibit insulin synthesis and secretion, highlighting these cells as an important target for the negative metabolic actions of glucocorticoids (Delaunay *et al.* 1999). Despite this, the possible sex-dependent differences of HC and STZ on glucose homeostasis, and especially pancreatic islet histology and cell lineage, has not been fully investigated (Kaikaew *et al.* 2019).

Therefore, the present study aims to compare and contrast the metabolic responses and alterations of pancreatic islet histology in response to STZ and HC treatment in male and female transgenic Glu^CreERT2^/Rosa26-eYFP mice. Importantly, use of Glu^CreERT2^/Rosa26-eYFP mice permits for investigation of the possible sex-dependent effects on pancreatic alpha-cell lineage and related alpha-to-beta-cell transdifferentiation, that is now known to be important for the development and progression of diabetes (Saleh *et al.* 2021). In that regard, the primary focus of the work was assessment of changes in pancreatic islet morphology, as well as alpha and beta islet cell populations, in male and female mice treated with STZ or HC. This is the first study to directly compare the sex-specific impact of STZ and HC on pancreatic islet cell morphology, as well as to investigate the impact of these diabetogens on islet cell transdifferentiation events in both sexes.

## Methods

### Animals

Transgenic Glu^CreERT2^/Rosa26-eYFP C57BL/6 mice were bred in house within the Biomedical and Behavioural Research Unit (BBRU) at Ulster University, Coleraine. These mice express an inducible eYFP reporter specifically in cells expressing the glucagon gene and have previously been fully characterised (Campbell *et al.* 2020). For experiments, mice were individually housed with *ad libitum* access to standard chow and water in a temperature controlled, 12-h light:12-h darkness cycle environment. The following experiments were approved by Ulster University Animal Welfare and Ethics Review Committee (AWERB) and conducted in line with the UK Animals (Scientific Procedures) Act 1986. In brief, 14-week-old male and female mice (*n* = 7 mice/group) received either saline control (0.9% NaCl), STZ (S0130, Sigma-Aldrich) or HC (H0888, Sigma-Aldrich) to induce insulin deficiency or insulin resistance, respectively, as detailed below. One week prior to administration of STZ or HC, all mice were administered a single i.p. dose of tamoxifen (7 mg/mouse, T2859, Sigma-Aldrich) to induce eYFP expression. To provoke insulin deficiency, STZ (50 mg/kg, freshly dissolved in citrate buffer) was administered by intraperitoneal (i.p.) injection on 5 consecutive days, with overt hyperglycaemia observed 5 days following the last injection. For insulin resistance, HC (70 mg/kg, dissolved in PBS) was administered by daily i.p. injections for 10 consecutive days. At regular intervals, body weight, non-fasting glucose and energy intake were recorded. Energy intake was assessed by weighing the food hopper of individually caged mice at regular timepoints. All experiments were terminated on day 11 with appropriate collection of blood and pancreatic tissues as described below.

### Biochemical analyses

Blood glucose was measured from tail vein blood using an Ascensia Contour blood glucometer. Terminal blood samples were collected in heparin/fluoride-coated microcentrifuge tubes and centrifuged for 10 min at 1500 ***g***. Separated plasma was then stored at −70℃ until assessment of insulin concentrations. At termination, pancreatic tissues were excised, divided longitudinally, and processed for either determination of pancreatic hormone content following acid/ethanol protein extraction or fixed in 4% paraformaldehyde for 48 h at 4℃ for histological analysis. Pancreatic and plasma insulin content were determined by radioimmunoassay (Flatt & Bailey 1981), whilst total protein content was assessed using Bradford reagent.

### Immunohistochemistry

Fixed tissue was embedded into paraffin wax blocks and sectioned at 5 µm slices using a microtome (Shandon Finesse 325, Thermo Scientific), and sections selected at intervals of every ten sections. Immunohistochemistry was conducted to assess islet morphology, cellular proliferation and apoptosis rates as well as alpha-cell transdifferentiation. In brief, slides were dewaxed in xylene and rehydrated in an alcohol gradient (100–50% EtOH). Antigen retrieval was carried out in citrate buffer (pH 6, 90℃), followed by blocking in 4% BSA solution. Slides were then incubated with primary antibodies (Table 1) overnight at 4℃, prior to rinsing in PBS and incubation with the appropriate fluorescently conjugated secondary antibodies (Table 1). Slides were also incubated with DAPI before mounting and imaging using a fluorescent microscope (Olympus model BX51) fitted with DAPI (350 nm), FITC (488 nm) and TRITC (594 nm) filters and a DP70 camera adapter system.

**Table 1:** Target, host, fluorophore, dilution, supplier and combinations employed of primary and secondary antibodies employed for immunohistochemistry.

Primary antibodies				
Target	Host	Fluorophore	Dilution	Supplier
Insulin	Mouse	N/A	1:400	Abcam (ab6995)
Glucagon	Rabbit	N/A	1:1000	Abcam (ab92517)
Glucagon^a^	Guinea pig	N/A	1:400	Raised in-house (PCA2/4)
GFP	Goat	N/A	1:500	Abcam (ab5450)
Ki-67	Rabbit	N/A	1:500	Abcam (ab15580)
**Secondary antibodies**				
**Target**	**Host**	**Fluorophore**	**Dilution**	**Supplier**
Mouse	Donkey	Alexa Fluor 594	1:400	Invitrogen (A-21203)
Mouse	Donkey	Alexa Fluor 488	1:400	Invitrogen (A-21202)
Rabbit	Donkey	Alexa Fluor 594	1:400	Invitrogen (A-21207)
Rabbit	Donkey	Alexa Fluor 488	1:400	Invitrogen (A-21206)
Goat	Donkey	Alexa Fluor 488	1:400	Invitrogen (A-11055)
Guinea pig	Goat	Alexa Fluor 488	1:400	Invitrogen (A-11073)

^a^To overcome species cross-reactivity when assessing alpha-cell proliferation, the guinea pig anti-glucagon antibody was employed for these experiments. For all other assessments of glucagon staining, the rabbit anti-glucagon antibody was employed.

### Image analysis

ImageJ software was used to analyse islet morphology, employing the ‘closed polygon’ tool to identify regions of insulin-positive and glucagon-positive staining areas to define beta- and alpha-cell areas, respectively (Khan *et al.* 2016). To quantify alpha- and beta-cell apoptosis and proliferation rates, the number of insulin or glucagon positive cells expressing TUNEL or Ki-67, respectively, were quantified and expressed as a percentage of total insulin/glucagon positive cells. To assess effects on alpha-cell lineage, YFP was detected with a goat anti-GFP antibody (Table 1), which is reactive against all variants of *Aequorea victoria* GFP, including YFP. In this regard, cells expressing both glucagon and GFP were considered ‘mature alpha cells’, whilst cells expressing GFP but lacking in glucagon were termed ‘dedifferentiated alpha-cells’ (Lafferty *et al.* 2021). In addition, islet cells expressing GFP together with insulin were deemed to have undergone alpha-to-beta-cell transdifferentiation.

### Statistics

Results were analysed using GraphPad PRISM (version 8.0) software, with data presented as mean ± S.E.M. Comparative analyses between groups were carried out using a one-way or two-way ANOVA, as appropriate, utilising a Bonferroni *post hoc* test for multiple comparisons between treatment groups and sex. In this respect, the *P*-values for interaction between treatment and sex fell between 0.1 and 1.0 with analysis subsequently progressing to *post hoc* tests to derive which groups have differences for each individual factor studied. Results were deemed significant if *P* < 0.05.

## Results

### Effects of STZ and HC on circulating glucose, plasma and pancreatic insulin, glucose tolerance, body weight and food intake in male and female mice

**Figure 1:**
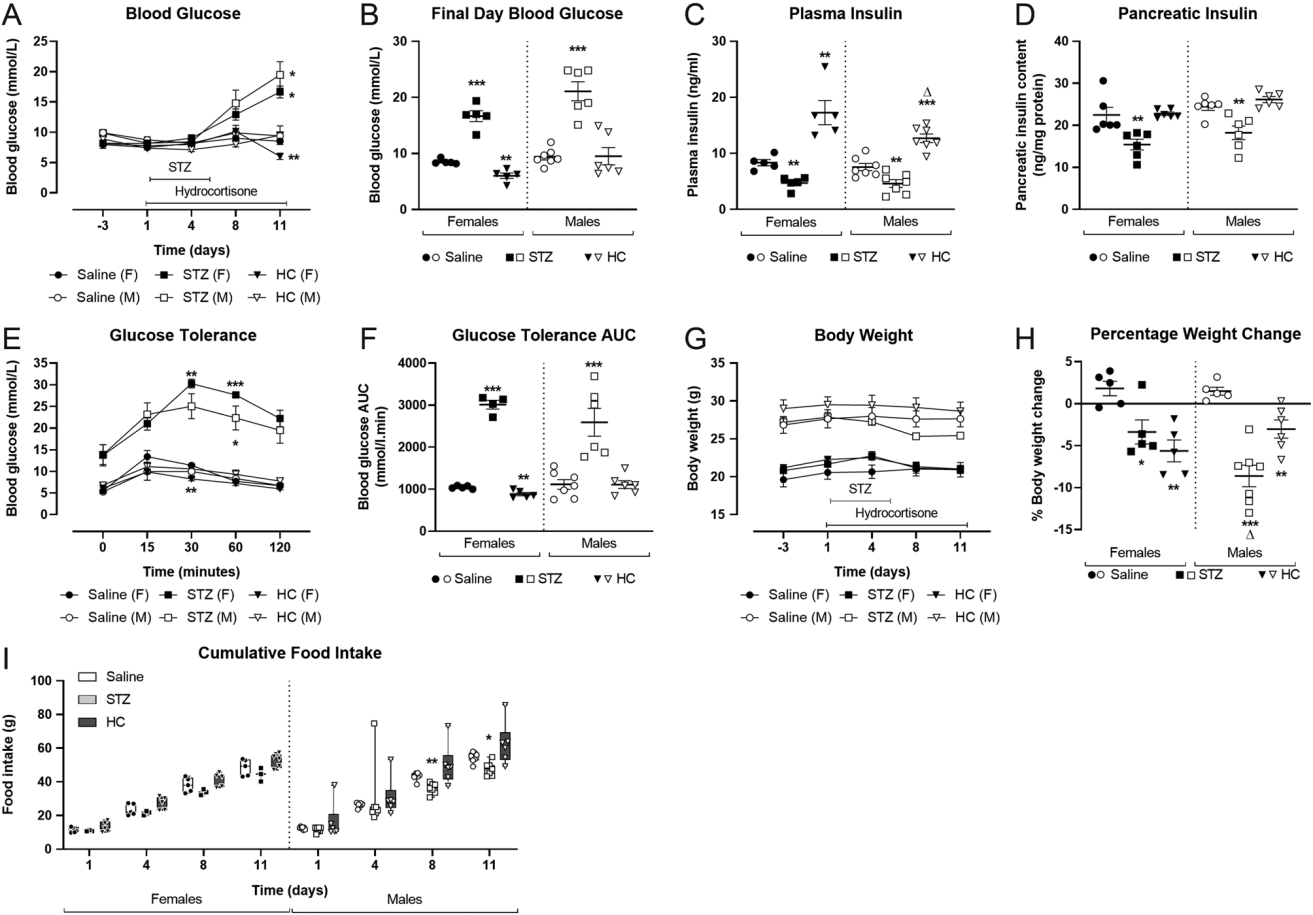
Effects of STZ or HC treatment on female and male Glu^CreERT2^/Rosa26-eYFP mice. (A, B) blood glucose, (C) plasma insulin, (D) pancreatic insulin, (E, F) glucose tolerance, (G) body weight, (H) percentage weight change and (I) cumulative food intake. Mice received five daily doses of streptozotocin (50 mg/kg, i.p.) or 10 daily doses of hydrocortisone (70 mg/kg, i.p.) with variables measured during (A, G, I) this period or on day 11 (B–F, H). All values represent mean ± s.e.m. for seven mice. *P < 0.05, **P < 0.01, ***P < 0.001 compared to saline controls. Δ*P* < 0.05 compared to respective female mice under the same treatment regimen. (B–D, F, H) Datasets were analysed using a one-way ANOVA with Bonferroni *post hoc* test or (A, E, G, I) using a two-way ANOVA with a Bonferroni *post hoc* test.

Male and female mice treated with multiple low-dose STZ developed comparable hyperglycaemia (*P* < 0.001, Fig. 1A and B), in conjunction with reduced plasma and pancreatic insulin concentrations (*P* < 0.01, Fig. 1C and D) and impaired glucose tolerance (*P* < 0.001, Fig. 1E and F) when compared to control mice. As expected, control male mice had elevated body weight when compared to females (26.8 ± 1.1 vs 19.6 ± 0.9 g, respectively; *P* < 0.01), with all STZ-treated mice presenting with reduced body weight (*P* < 0.05 to *P* > 0.001, Fig. 1G and H), and interestingly this associated with a small but significant reduction in food intake in male, but not female, mice (*P* < 0.05, Fig. 1I). Administration of HC reduced non-fasting blood glucose in female mice on day 11 (*P* < 0.01, Fig. 1B), but this effect was not apparent in male mice (Fig. 1B). Moreover, glucose tolerance was slightly improved in female, but not male, HC-treated mice (*P* < 0.01, Fig. 1E and F). As expected, plasma insulin levels were elevated in all HC-treated mice (*P* < 0.01–0.001, Fig. 1C), but significantly less so in males (*P* < 0.05, Fig. 1C), with no obvious changes in pancreatic insulin content (Fig. 1D). Body weights of all HC mice were reduced when compared to controls (*P* < 0.05–0.01, Fig. 1G and H), but this occurred in the absence of changes in food intake (Fig. 1I).

### Effects of STZ and HC on pancreatic islet histology in male and female mice

**Figure 2:**
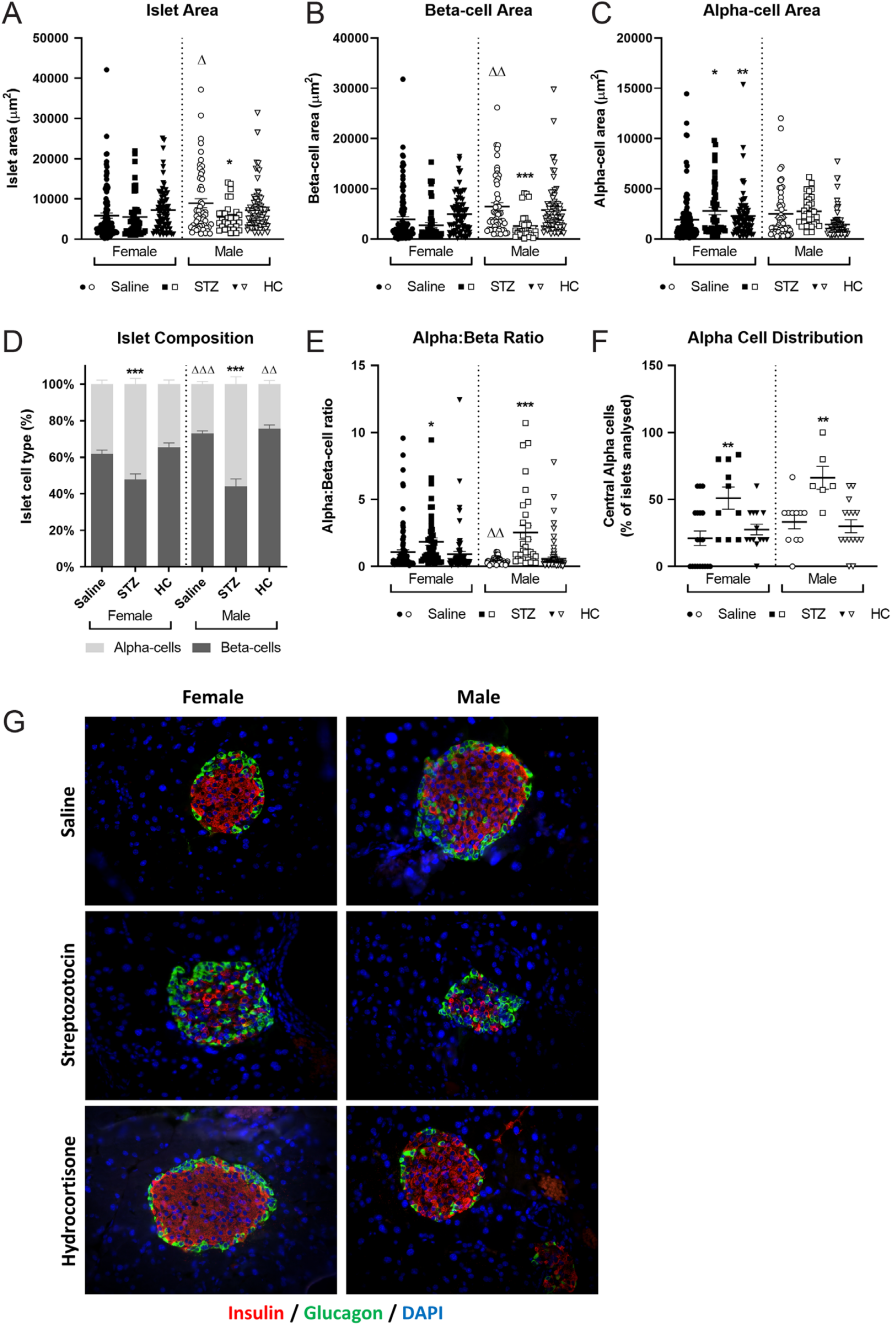
Effects of STZ or HC treatment on pancreatic islet morphology in female and male Glu^CreERT2^/Rosa26-eYFP mice. (A) Islet area, (B) beta-cell area, (C) alpha-cell area, (D) alpha:beta ratio, (E) alpha-cell distribution and (F) islet composition were assessed on day 11. (G) Representative images of islets from each group highlighting insulin and glucagon staining. All values represent mean ± s.e.m., with >50 islets analysed per group. *P < 0.05, **P < 0.01, ***P < 0.001 compared to saline controls. Δ*P* < 0.05, ΔΔ*P* < 0.01 compared to respective female mice under the same treatment regimen. Datasets were analysed using a one-way ANOVA with Bonferroni *post hoc* test. A full colour version of this figure is available at https://doi.org/10.1530/JOE-23-0174.

Islet size and composition varied with sex, with healthy female mice having reduced islet area when compared to their male counterparts (*P* < 0.05–0.01, Fig. 2A) that were composed of fewer beta cells (*P* < 0.001, Fig. 2B, C, D and E). When treated with low-dose STZ, male mice presented with reduced islet (*P* < 0.05, Fig. 2A) and beta-cell areas (*P* < 0.001, Fig. 2B), an effect that was not observed in females (Fig. 2B). STZ treatment elevated alpha-cell area in female mice (*P* < 0.05, Fig. 2C), with alpha:beta ratio greatly increased in both sexes (*P* < 0.05–0.001, Fig. 2E). In addition, the number of islets with centrally distributed alpha cells was notably increased in STZ-treated mice of both sexes (*P* < 0.01, Fig. 2F). HC treatment had no discernible impact on pancreatic islet morphology in either male or female mice, with the exception that female mouse islets were composed of more alpha cells than their HC treated male counterparts (*P* < 0.01, Fig. 2D). Representative images of stained islets are shown in Fig. 2G.

### Effects of STZ and HC on pancreatic islet cell turnover in male and female mice

**Figure 3:**
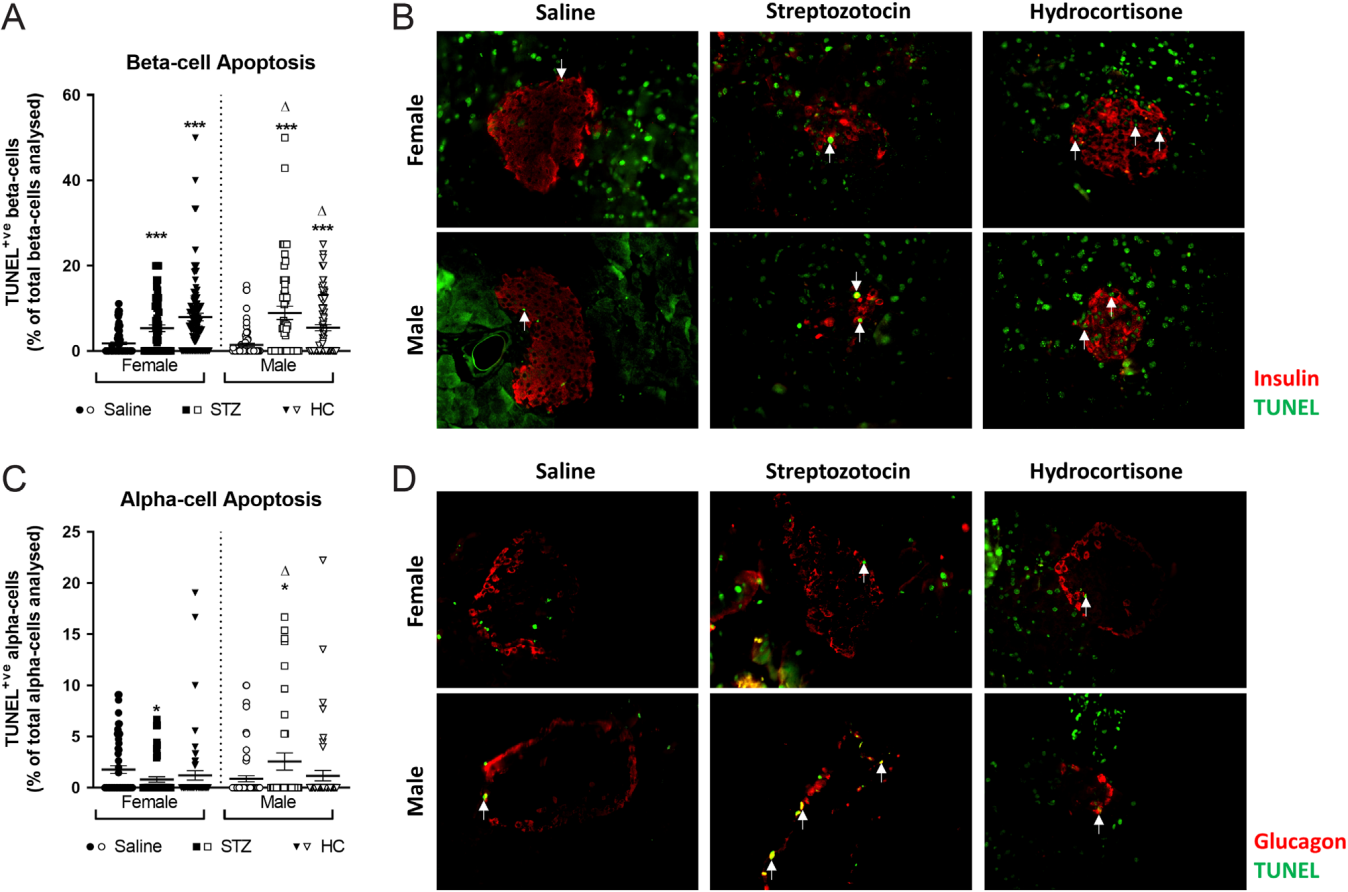
Effects of STZ or HC treatment on islet cell apoptosis in female and male Glu^CreERT2^/Rosa26-eYFP mice. (A) Beta-cell apoptosis and (B) alpha-cell apoptosis were assessed on day 11. Representative images of islets from each group showing (C) insulin and TUNEL or (D) glucagon and TUNEL co-staining. All values represent mean ± s.e.m., with >50 islets analysed per group. *P < 0.05, ***P < 0.001 compared to saline controls. Δ*P*< 0.05 compared to respective female mice under the same treatment regimen. Datasets were analysed using a one-way ANOVA with Bonferroni *post hoc* test. A full colour version of this figure is available at https://doi.org/10.1530/JOE-23-0174.

**Figure 4:**
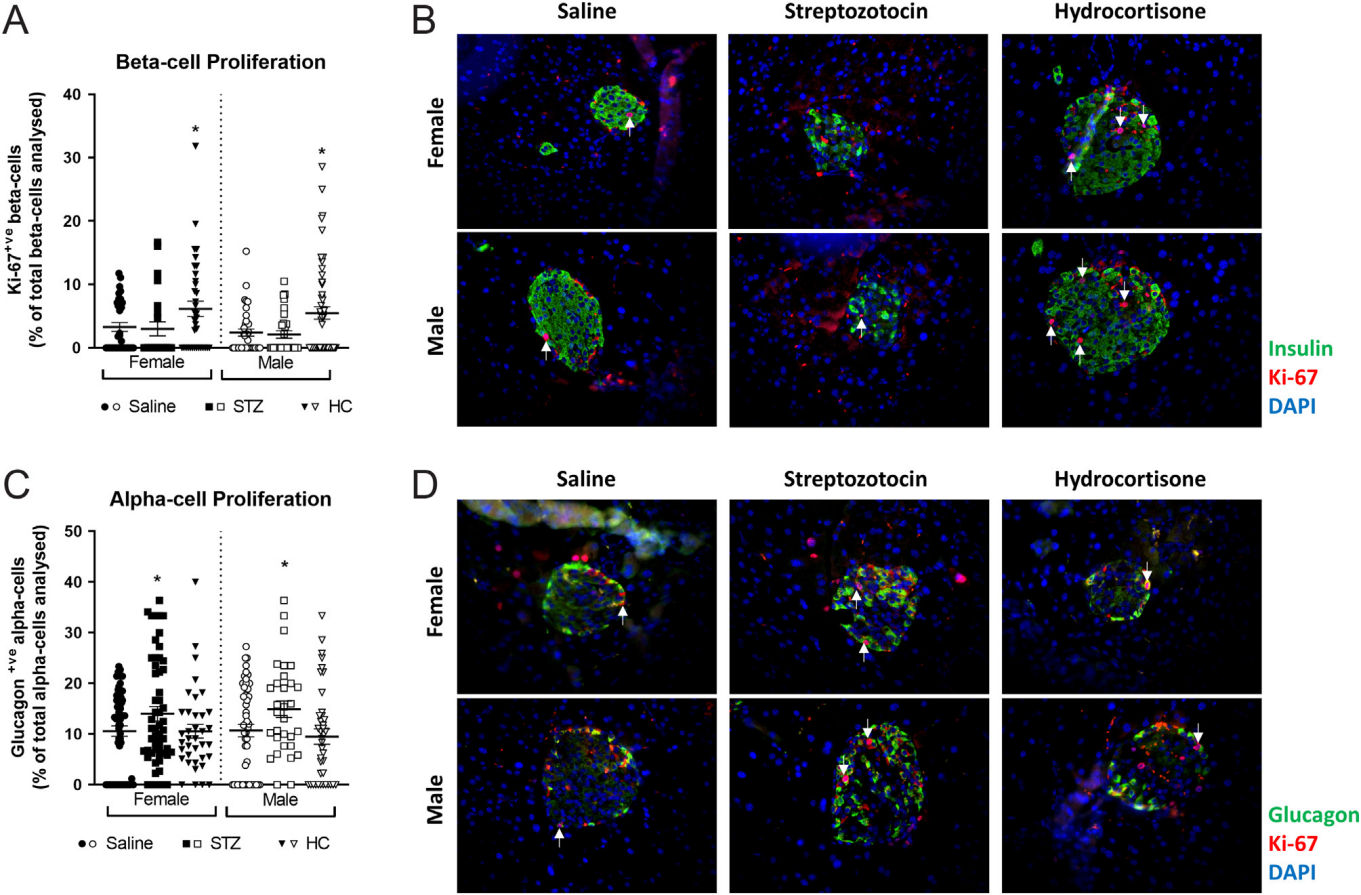
Effects of STZ or HC treatment on islet cell proliferation in female and male Glu^CreERT2^/Rosa26-eYFP mice. (A) Beta-cell proliferation and (B) alpha-cell proliferation were assessed on day 11. Representative images of islets from each group showing (C) insulin and Ki-67 or (D) glucagon and Ki-67 co-staining. All values represent mean ± s.e.m., with >50 islets analysed per group. *P < 0.05 compared to saline controls. Datasets were analysed using a one-way ANOVA with Bonferroni *post hoc* test. A full colour version of this figure is available at https://doi.org/10.1530/JOE-23-0174.

**Figure 5:**
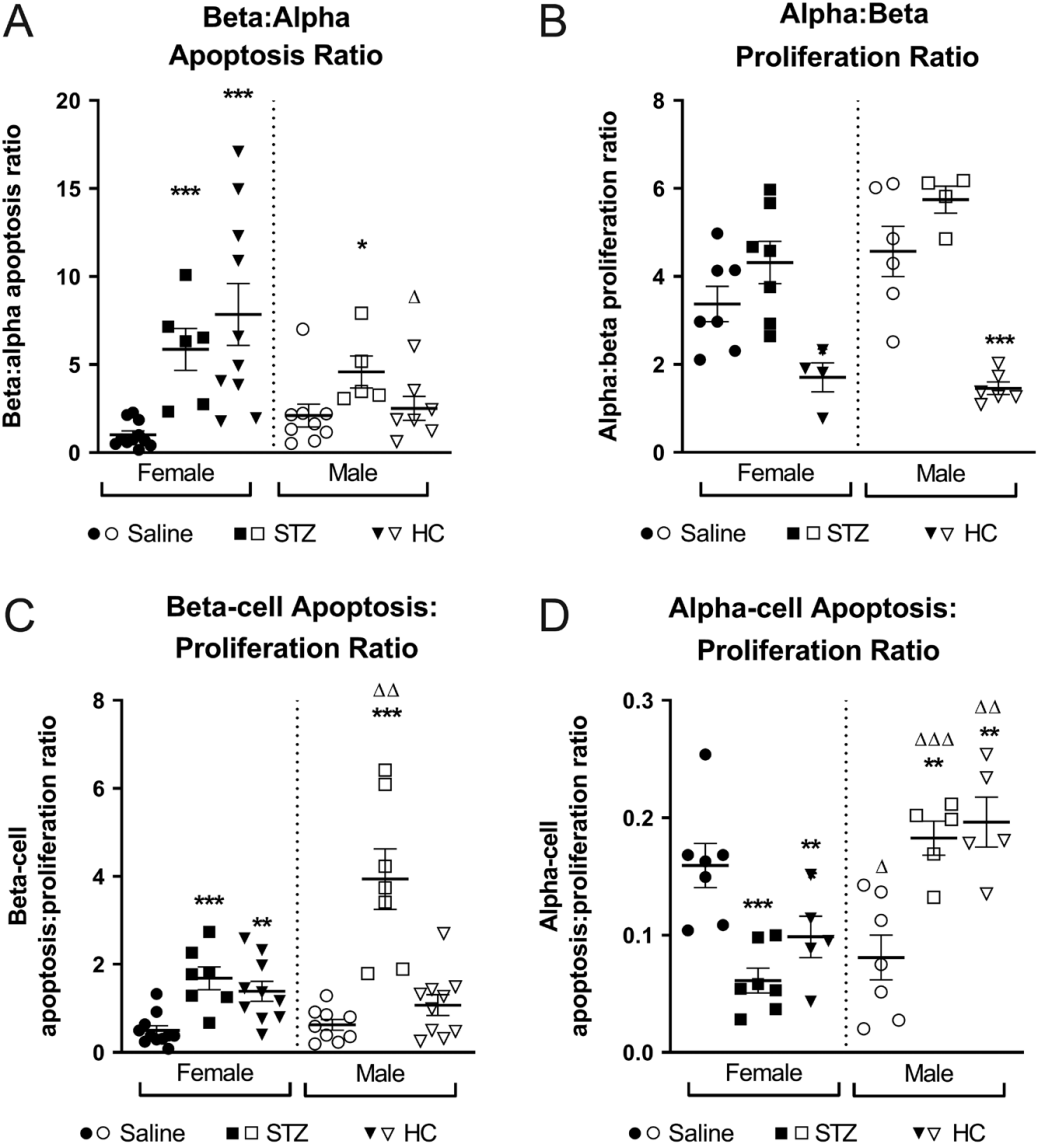
Effects of STZ or HC treatment on islet cell proliferation and apoptosis ratios in female and male Glu^CreERT2^/Rosa26-eYFP mice. (A) beta:alpha apoptosis ratio (B) alpha:beta proliferation ratio, (C) beta-cell apoptosis:proliferation ratio and (D) alpha-cell apoptosis:proliferation ratio were both determined on day 11. All values represent mean ± s.e.m., with >50 islets analysed per group. *P < 0.05, **P < 0.01, ***P < 0.001 compared to saline controls. Δ*P* <0.05, ΔΔ*P* < 0.01, ΔΔΔ*P* < 0.001 compared to respective female mice under the same treatment regimen. Datasets were analysed using a one-way ANOVA with Bonferroni *post hoc* test.

Multiple low-dose STZ treatment increased beta-cell apoptosis in all mice (*P* < 0.001, Fig. 3A and B), but this was significantly more apparent in males (*P* < 0.05, Fig. 3A). Likewise, alpha-cell apoptosis was elevated in male STZ-treated mice (*P* < 0.05, Fig. 3C and D), with STZ female mice presenting with reduced alpha-cell apoptotic rates (*P* < 0.05, Fig. 3C). Beta-cell proliferation was unaffected by STZ (Fig. 4A and B), whilst alpha-cell proliferation was significantly increased in all STZ mice (*P* < 0.05, Fig. 4C and D). Further analyses of islet cell turnover rates confirmed that STZ selectively targeted beta cells for apoptosis regardless of the sex of the mouse (Fig. 5A), with no impact on proliferation frequency of these cells (Fig. 5B). In all STZ-treated mouse islets, beta-cell apoptosis significantly outweighed proliferation (*P* < 0.001, Fig. 5C), but the effect was more prominent in male mice (*P* < 0.01, Fig. 5C). Interestingly, alpha-cell apoptosis also predominated over proliferation in STZ-treated male mice (*P* < 0.01 Fig. 5D), but the opposite was observed in female mice (*P* < 0.001 Fig. 5D).

HC treatment elicited an increase in beta-cell apoptosis (*P* < 0.001, Fig. 3A and B) and proliferation (*P* < 0.05, Fig. 4A and B) in all mice. However, beta-cell apoptosis rates were elevated in male as compared to female HC-treated mice (*P* < 0.05, Fig. 3A). Alpha-cell apoptosis and proliferation were unaffected by HC treatment and not different between sexes (Fig. 3C and 4C). Accordingly, the beta:alpha cell apoptosis ratio was enhanced in female HC treated mice when compared to controls and their male counterparts (*P* < 0.05–0.001, Fig. 5A). In all HC treated mice, alpha:beta cell apoptosis ratio was consistently reduced when compared to control mice (*P* < 0.05–0.001, Fig. 5B). On specific examination of alpha and beta cells, apoptosis outweighed proliferation within beta cells of female HC mice, whilst the opposite was true in alpha cells, when compared to control mice (*P* < 0.05–0.01, Fig. 5C and D). In male HC mice, there was no difference in the balance of apoptosis and proliferation rates in beta cells (Fig. 5C), but apoptosis was elevated within alpha cells (*P* < 0.01, Fig. 5D). In addition, female HC mice had a significantly decreased apoptosis:proliferation ratio in alpha cells when compared to males (*P* < 0.01, Fig. 5D).

### Effects of STZ and HC on pancreatic islet cell lineage in male and female mice

**Figure 6:**
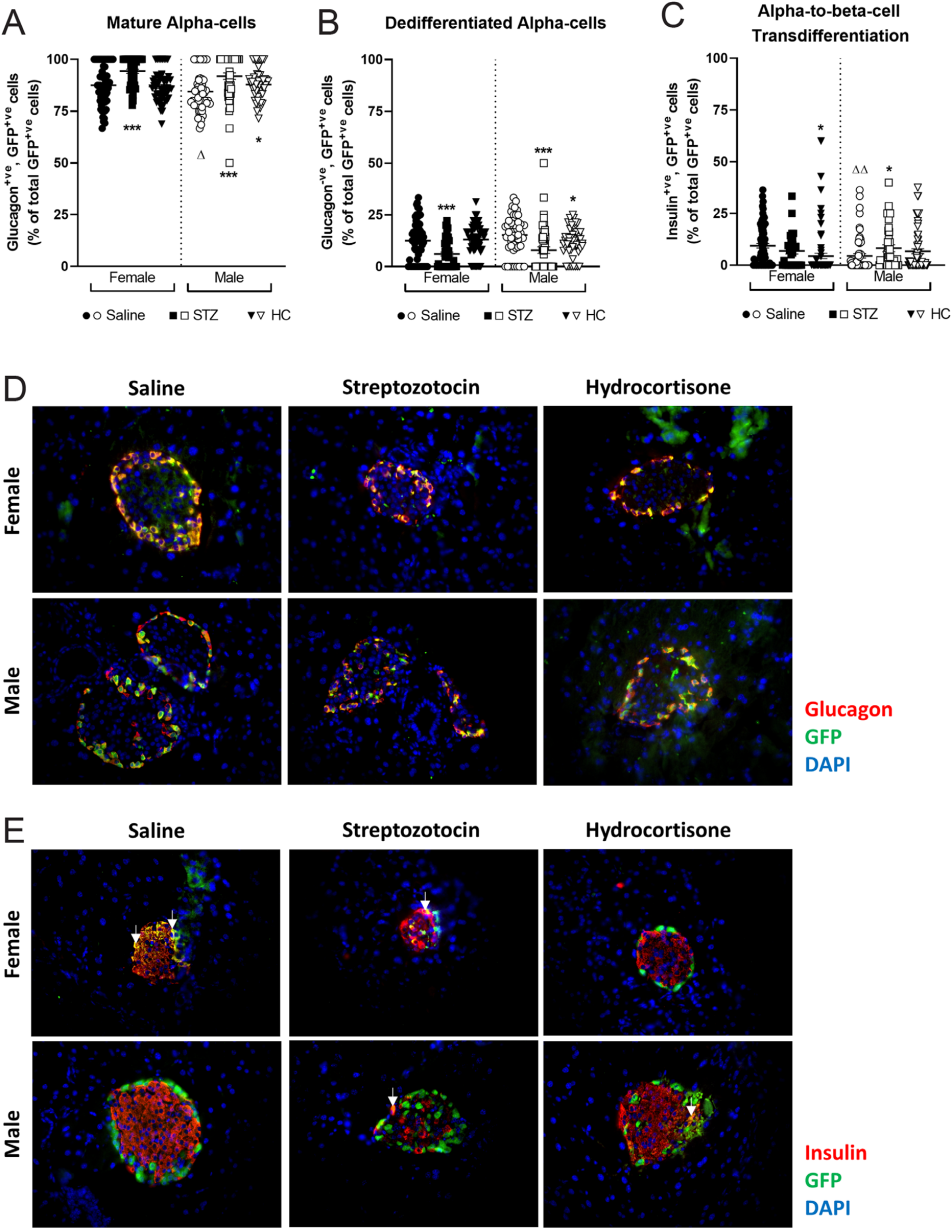
Effects of STZ or HC treatment on alpha-cell lineage in female and male Glu^CreERT2^/Rosa26-eYFP mice. (A) Mature alpha cells, (B) dedifferentiated alpha cells and (C) alpha-to-beta-cell transdifferentiation were assessed on day 11. All values represent mean ± s.e.m., with >50 islets analysed per group. Representative images of islets from each group of mice stained for glucagon (D) or insulin (E) alongside GFP and DAPI are also shown. *P < 0.05, ***P < 0.001 compared to saline controls. Δ*P* < 0.05, ΔΔ*P* < 0.01 compared to respective female mice under the same treatment regimen. Datasets were analysed using a one-way ANOVA with Bonferroni *post hoc* test. A full colour version of this figure is available at https://doi.org/10.1530/JOE-23-0174.

Lineage tracing of alpha cells, utilising GFP staining, revealed that STZ treatment increased the percentage of GFP-positive cells co-expressing glucagon (mature alpha cells) as well as decreasing numbers of glucagon negative, GFP-positive (dedifferentiated alpha cells) islet cells independent of mouse sex (*P* < 0.001, Fig. 6A and B). HC treatment produced a similar increase in cells co-expressing both glucagon and GFP and decrease in cells expressing GFP without glucagon, but interestingly only in male mice (*P* < 0.01, Fig. 6A and B). However, it should also be noted that saline-treated female control mice had a greater percentage of mature alpha cells than corresponding males (*P* < 0.05, Fig. 6A). Healthy female mice displayed higher numbers of GFP-positive cells co-expressing insulin (alpha-to-beta-cell transdifferentiation) when compared to males (*P* < 0.01, Fig. 6C). In female mice, only HC treatment reduced the number of GFP cells co-expressing insulin (*P* < 0.05, Fig. 6C), whilst STZ treatment increased the number of GFP positive cells expressing insulin only in male mice (*P* < 0.05, Fig. 6C). Representative images of islets from each group of mice stained for glucagon or insulin alongside GFP are depicted in Fig. 6D and E, respectively.

## Discussion

The present study explored how sex impacts pancreatic islet adaptive responses to STZ and HC administration in mice. As expected, STZ and HC elicited characteristic metabolic dysregulation, which was not dependent on the sex of the animal (Tanday *et al.* 2020*a*, Daniels Gatward *et al.* 2021). However, subtle sex-specific differences were observed at the level of the endocrine islet that merit further exploration. This serves to emphasise the importance of understanding the implications of sex on diabetic pathophysiology, and potential related influences on drug discovery programs.

Specifically, in the current setting, male mice displayed typical STZ-induced beta-cell damage, with associated severe hyperglycaemia and weight loss, akin to previous observations (Tanday *et al.* 2020*a*) and likely related to an increased proportion of beta cells in male mice (Yokomizo *et al.* 2014). There is a belief that female mice are more tolerant to the detrimental effects of STZ than males (Ariza *et al.* 2014, Chandramouli *et al.* 2018, Kim *et al.* 2020). In that respect, several studies have already investigated the sex-specific effects of STZ treatment in mice, with a general consensus that the diabetogen causes more detrimental effects on pancreatic islet morphology in male than female mice (Kolb 1987, Abildgaard *et al.* 2020, Kim *et al.* 2020, 2023), although this has been debated (Plesner *et al.* 2014). Mechanistically, increased STZ resistance in female mice has been demonstrated to be related to higher oestrogen levels, with oestrogen known to suppress inflammation (Abildgaard *et al.* 2020), protect against inflammatory cytokine insult (Sachs *et al.* 2020), promote misfolded protein degradation (Xu *et al.* 2018) and enhance glucose-stimulated insulin secretion (Tiano & Mauvais-Jarvis 2012). Indeed, upon ovariectomy, female mice become more susceptible to STZ with similar metabolic dysregulation as observed in male mice (Kim *et al.* 2023). In harmony with this, ovariectomised female mice treated with oestradiol present with an improved protection against the harmful effects of STZ (Li *et al.* 2018). Our data would support some inherent protection from the islet cell destructive effects of STZ in female mice, but importantly both male and female Glu^CreERT2^/Rosa26-eYFP mice presented with blood glucose levels in excess of 15 mmol/L following STZ administration, combined with severely impaired glucose tolerance and reduced plasma and pancreatic insulin. Indeed, the only assessed metabolic parameter where STZ was more harmful to male over female mice was body weight loss, which appeared to be linked to decreased food intake. However, this observation does need to be considered in light of the initial increased body weight of male mice (de Souza *et al.* 2022).

In keeping with this, pancreatic islets from male mice were larger than females, which may represent another factor as to why male mice displayed more obvious STZ-induced reductions in islet and beta-cell areas. In some accord, female STZ mice exhibited less pronounced increases in beta-cell apoptosis, perhaps linked to protective oestrogen actions (Abildgaard *et al.* 2020). This may be particularly relevant given the perceived importance of modulation of programmed beta-cell death for both the onset of diabetes (Eizirik *et al.* 2020), as well as the effectiveness of some antidiabetic therapies including GLP-1 receptor agonists (Kapodistria *et al.* 2018, Díaz-Megido & Thomsen 2023). In that regard, similar investigations in ovariectomised female mice with depleted oestrogen levels would be of interest. It is perhaps also worth noting that a single low dose tamoxifen injection was employed to induce Cre–lox recombination in all our Glu^CreERT2^/Rosa26-eYFP transgenic mice, but this is a commonly employed tool with minimal adverse effects (Lafferty *et al.* 2021) and only prolonged exposure at elevated doses should directly impact metabolic state (Ceasrine *et al.* 2019). Thus, whilst low dose tamoxifen has been suggested to alter metabolism in mice, this earlier investigation employed either repeated administration at a dose more than three times our selected 7 mg/kg tamoxifen dose, or a single tamoxifen injection some seven times greater than this (Zhao *et al.* 2020). That said, we are unable to completely rule out an impact of the single low-dose tamoxifen injection on metabolism and islet morphology, despite the oestrogen receptor modulator being delivered 18 days prior to pancreatic tissue extraction, since persistent effects of a single tamoxifen injection are reported under certain environments (Patel *et al.* 2017, Stout *et al.* 2021). Accordingly, Glu^CreERT2^/Rosa26-eYFP transgenic mice require tamoxifen administration to study changes in islet cell lineage, but it would be interesting to examine the impact of STZ and HC on the other aspects of pancreatic islet architecture assessed within the current setting using wildtype male and female mice. Unfortunately, such additional studies are outside the scope of the current work, however this factor should not be totally discounted when interpreting our datasets.

Although the impact of chemical diabetogens on beta-cell health is often the primary consideration in terms of modelling the phenotype in rodents, effects on the alpha cell merit further contemplation (Gannon *et al.* 2018). This seems particularly relevant given recent knowledge on the plasticity of mature islet cells and ability of alpha cells to function as progenitors for insulin positive beta cells through distinct lineage alteration (Tanday *et al.* 2020*b*). Moreover, alpha cells are innately more resistant to oxidative stress and induction of apoptosis than beta cells (Eizirik *et al.* 2023). Indeed, there is a suggestion that alpha cells could represent a viable direct target for diabetes therapies (Klempel *et al.* 2022). Notably, in the current study female mouse islets were composed of more alpha cells than males, with no obvious difference in turnover rates. Thus, our observations of increased alpha-to-beta-cell transdifferentiation in female mice may be related to this phenomenon as an inherent adaptation to increased alpha-cell area, but this still needs to be confirmed. A small study in 52 non-diabetic subjects reports that alpha-cell mass is not different between men and women (Henquin & Rahier 2011), whereas others suggest females have slightly more beta cells than males (Marchese *et al.* 2015), highlighting some uncertainty in this regard. However, such observations also need to be considered with respect to the apparent differences between human and rodent alpha-cell signalling and function (Moede *et al.* 2020).

Interestingly, in response to multiple low-dose STZ treatment, male and female mice had opposing effects on alpha-cell apoptotic rates, but similar increases in proliferation, resulting in elevated alpha-cell area especially in female mice. Alpha-to-beta-cell transdifferentiation was increased in male STZ mice, likely as an adaptive response to help preserve beta-cell mass. Thus, in the absence of oestrogen signalling that possesses independent beta-cell protective actions (Kim *et al.* 2023), islet cell lineage alteration in the face of a specific beta-cell insult could be paramount. Whether oestrogen could be exploited as an antidiabetic therapy is debatable, and detrimental off-target side effects may ultimately limit overall effectiveness (Sjögren *et al.* 2016). However, approaches utilising GLP-1/oestrogen conjugates to allow for more targeted oestrogen delivery are encouraging (Schwenk *et al.* 2014, Sachs *et al.* 2020).

Insulin resistance is a major risk factor for T2D and can be modelled preclinically by sustained administration of glucocorticoids. Whilst females appear to have inherent protection against developing insulin resistance (Aldhoon-Hainerová *et al.* 2014), this is absent upon menopause (Janssen *et al.* 2008). In keeping with this, the risk of insulin resistance increases in females when the menopause is surgically induced (Christakis *et al.* 2020) and decreases with hormone replacement therapy (Salpeter *et al.* 2006). Intriguingly, in our model, HC treatment resulted in mild insulin resistance typified by elevated plasma insulin levels, especially in female mice, with no real alteration of glucose homeostasis. Morphologically, pancreatic islets were largely unaltered in HC treated mice, that perhaps contrasts with other reports (Vasu *et al.* 2014, Mohan *et al.* 2022) but agrees with our observations of equivalent increases in beta-cell proliferation and apoptosis rates that was unrelated to sex. Interestingly, despite a putative protective effect of oestrogen on beta-cell survival (Sachs *et al.* 2020), female HC mice displayed particularly high rates of beta-cell apoptosis. Androgens have been shown to sensitise mice to glucocorticoid-induced insulin resistance, and it may be interesting to assess the impact of orchidectomy in our male HC mice (Gasparini *et al.* 2019). Perhaps most fascinating was the decrease in alpha-to-beta-cell lineage switch in female HC mice, suggesting that at least in female mice this pathway is less important for regulating beta-cell mass in the face of insulin resistance. That said, male HC mice had increased numbers of mature, and decreased numbers of dedifferentiated alpha cells, suggesting specific islet cell lineage changes that merit more in-depth assessment. Collectively, alpha-to-beta-cell transdifferentiation was elevated in male STZ mice, but decreased by HC administration in female mice, possibly reflecting both sex-specific actions and the contrasting aetiologies of these rodent models (Daniels Gatward *et al.* 2021).

In conclusion, STZ- and HC-induced insulin deficiency or resistance, respectively, in Glu^CreERT2^/Rosa26-eYFP transgenic mice results in characteristic metabolic outcomes that are similar in male and female mice. However, there are notable sex-specific differences in associated adaptive responses within the pancreatic islets of these mice. We have described alterations in both islet morphology as well as cellular turnover and transdifferentiation rates between sexes, that merit further consideration and should be remembered when extrapolating observations from males to females.

## Declaration of interest

All authors declare no conflict of interest.

## Funding

These studies were supported by Ulster University Research Funding support and award of an Ulster University Vice Chancellors Research Studentship to VD. RCM is supported by an RD Lawrence Fellowship awarded by Diabetes UK.

## Data availability

The authors declare that the data supporting the findings of this study are available within the article. Any additional raw data supporting the conclusions of this article will be made available by the lead author, without undue reservation.

## Author contribution statement

NT, RCM, PRF and NI conceived/designed the study. NI and NT drafted the manuscript. NT, AC-P, KS and VD participated in the conduct/data collection and analysis and interpretation of data. All authors revised the manuscript critically for intellectual content and approved the final version of the manuscript.

## References

[bib1] Abildgaard J Tingstedt J Zhao Y Hartling HJ Pedersen AT Lindegaard B & Dam Nielsen S 2020 Increased systemic inflammation and altered distribution of T-cell subsets in postmenopausal women. PLoS One 15 e0235174. (10.1371/journal.pone.0235174) 32574226 PMC7310708

[bib2] Aldhoon-Hainerová I Zamrazilová H Dušátková L Sedláčková B Hlavatý P Hill M Hampl R Kunešová M & Hainer V 2014 Glucose homeostasis and insulin resistance: prevalence, gender differences and predictors in adolescents. Diabetology and Metabolic Syndrome 6 100. (10.1186/1758-5996-6-100) 25419241 PMC4240882

[bib3] Ariza L Zaguirre M García M Blasco E Rabanal RM Bosch A & Otaegui PJ 2014 Hyperglycemia and hepatic tumors in ICR mice neonatally injected with streptozotocin. Lab Animal 43 242–249. (10.1038/laban.530) 24945225

[bib4] Bailey CJ & Matty AJ 1972 Glucose tolerance and plasma insulin of the rat in relation to the oestrous cycle and sex hormones. Hormone and Metabolic Research 4 266–270. (10.1055/s-0028-1094063) 4673608

[bib5] Campbell JR Martchenko A Sweeney ME Maalouf MF Psichas A Gribble FM Reimann F & Brubaker PL 2020 Essential role of syntaxin-binding protein-1 in the regulation of glucagon-like peptide-1 secretion. Endocrinology 161 bqaa039. (10.1210/endocr/bqaa039) 32141504 PMC7124137

[bib6] Ceasrine AM Ruiz-Otero N Lin EE Lumelsky DN Boehm ED & Kuruvilla R 2019 Tamoxifen improves glucose tolerance in a delivery-, sex-, and strain-dependent manner in mice. Endocrinology 160 782–790. (10.1210/en.2018-00985) 30759201 PMC6424092

[bib7] Chandramouli C Reichelt ME Curl CL Varma U Bienvenu LA Koutsifeli P Raaijmakers AJA De Blasio MJ Qin CX Jenkins AJ, 2018 Diastolic dysfunction is more apparent in STZ-induced diabetic female mice, despite less pronounced hyperglycemia. Scientific Reports 8 2346. (10.1038/s41598-018-20703-8) 29402990 PMC5799292

[bib8] Christakis MK Hasan H de Souza LR & Shirreff L 2020 The effect of menopause on metabolic syndrome: cross-sectional results from the Canadian longitudinal study on aging. Menopause 27 999–1009. (10.1097/GME.0000000000001575) 32852451

[bib9] Daniels Gatward LF Kennard MR Smith LIF & King AJF 2021 The use of mice in diabetes research: the impact of physiological characteristics, choice of model and husbandry practices. Diabetic Medicine 38 e14711. (10.1111/dme.14711) 34614258

[bib10] de Souza GO Wasinski F & Donato J 2022 Characterization of the metabolic differences between male and female C57BL/6 mice. Life Sciences 301 120636. (10.1016/j.lfs.2022.120636) 35568227

[bib11] Delaunay F Khan A Cintra A Davani B Ling ZC Andersson A Ostenson CG Gustafsson J Efendic S & Okret S 1999 Pancreatic beta cells are important targets for the diabetogenic effects of glucocorticoids. Journal of Clinical Investigation 100 2094–2098. (10.1172/JCI119743) PMC508401 9329975

[bib12] Díaz-Megido C & Thomsen M 2023 Sex-dependent divergence in the effects of GLP-1 agonist exendin-4 on alcohol reinforcement and reinstatement in C57BL/6J mice. Psychopharmacology 240 1287–1298. (10.1007/s00213-023-06367-x) 37106129 PMC10172234

[bib13] Dirlewanger M Schneiter PH Paquot N Jequier E Rey V & Tappy L 2000 Effects of glucocorticoids on hepatic sensitivity to insulin and glucagon in man. Clinical Nutrition 19 29–34. (10.1054/clnu.1999.0064) 10700531

[bib14] Eizirik DL Pasquali L & Cnop M 2020 Pancreatic β-cells in type 1 and type 2 diabetes mellitus: different pathways to failure. Nature Reviews. Endocrinology 16 349–362. (10.1038/s41574-020-0355-7) 32398822

[bib15] Eizirik DL Szymczak F & Mallone R 2023 Why does the immune system destroy pancreatic β-cells but not α-cells in type 1 diabetes? Nature Reviews. Endocrinology 19 425–434. (10.1038/s41574-023-00826-3) 37072614

[bib16] FD A 1993 Guideline for the study and evaluation of gender differences in the clinical evaluation of drugs; notice. Federal Register 58 39406–39416. 11645233

[bib17] Flatt PR & Bailey CJ 1981 Abnormal Plasma Glucose and Insulin Responses in Heterozygous Lean (ob/+) mice. Diabetologia 20 573-577. (10.1007/bf00252768) 7026332

[bib18] Gannon M Kulkarni RN Tse HM & Mauvais-Jarvis F 2018 Sex differences underlying pancreatic islet biology and its dysfunction. Molecular Metabolism 15 82–91. (10.1016/j.molmet.2018.05.017) 29891438 PMC6066785

[bib19] Gasparini SJ Swarbrick MM Kim S Thai LJ Henneicke H Cavanagh LL Tu J Weber MC Zhou H & Seibel MJ 2019 Androgens sensitise mice to glucocorticoid-induced insulin resistance and fat accumulation. Diabetologia 62 1463–1477. (10.1007/s00125-019-4887-0) 31098671

[bib20] Hansen KB Vilsbøll T Bagger JI Holst JJ & Knop FK 2010 Reduced glucose tolerance and insulin resistance induced by steroid treatment, relative physical inactivity, and high-calorie diet impairs the incretin effect in healthy subjects. Journal of Clinical Endocrinology and Metabolism 95 3309–3317. (10.1210/jc.2010-0119) 20410219

[bib21] Henquin JC & Rahier J 2011 Pancreatic alpha cell mass in European subjects with type 2 diabetes. Diabetologia 54 1720–1725. (10.1007/s00125-011-2118-4) 21465328 PMC3110273

[bib22] Janssen I Powell LH Crawford S Lasley B & Sutton-Tyrrell K 2008 Menopause and the metabolic syndrome: the study of women's health across the nation. Archives of Internal Medicine 168 1568–1575. (10.1001/archinte.168.14.1568) 18663170 PMC2894539

[bib23] Kaikaew K Steenbergen J van Dijk TH Grefhorst A & Visser JA 2019 Sex difference in corticosterone-induced insulin resistance in mice. Endocrinology 160 2367–2387. (10.1210/en.2019-00194) 31265057 PMC6760317

[bib24] Kapodistria K Tsilibary EP Kotsopoulou E Moustardas P & Kitsiou P 2018 Liraglutide, a human glucagon‐like peptide‐1 analogue, stimulates AKT‐dependent survival signalling and inhibits pancreatic β‐cell apoptosis. Journal of Cellular and Molecular Medicine 22 2970–2980. (10.1111/jcmm.13259) 29524296 PMC5980190

[bib25] Kautzky-Willer A Leutner M & Harreiter J 2023 Sex differences in type 2 diabetes. Diabetologia 66 986–1002. (10.1007/s00125-023-05891-x) 36897358 PMC10163139

[bib26] Khan D Vasu S Moffett RC Irwin N & Flatt PR 2016 Islet distribution of peptide YY and its regulatory role in primary mouse islets and immortalised rodent and human beta-cell function and survival. Molecular and Cellular Endocrinology 436 102–113. (10.1016/j.mce.2016.07.020) 27465830

[bib27] Kim B Kim YY Nguyen PT Nam H & Suh JG 2020 Sex differences in glucose metabolism of streptozotocin-induced diabetes inbred mice (C57BL/6J). Applied Biological Chemistry 63 1–8. (10.1186/s13765-020-00547-5)

[bib28] Kim B Park ES Lee JS & Suh JG 2023 Outbred mice with streptozotocin-induced diabetes show sex differences in glucose metabolism. International Journal of Molecular Sciences 24 5210. (10.3390/ijms24065210) 36982285 PMC10049093

[bib29] Klempel N Thomas K Conlon JM Flatt PR & Irwin N 2022 Alpha-cells and therapy of diabetes: inhibition, antagonism or death? Peptides 157 170877. (10.1016/j.peptides.2022.170877) 36108978

[bib30] Kolb H 1987 Mouse models of insulin dependent diabetes: low‐dose streptozocin‐induced diabetes and nonobese diabetic (NOD) mice. Diabetes/Metabolism Reviews 3 751–778. (10.1002/dmr.5610030308) 2956075

[bib31] Lafferty RA Tanday N Moffett RC Reimann F Gribble FM Flatt PR & Irwin N 2021 Positive effects of NPY1 receptor activation on islet structure are driven by pancreatic alpha-and beta-cell transdifferentiation in diabetic mice. Frontiers in Endocrinology 12 633625. (10.3389/fendo.2021.633625) 33716983 PMC7949013

[bib32] Lenzen S 2008 The mechanisms of alloxan-and streptozotocin-induced diabetes. Diabetologia 51 216–226. (10.1007/s00125-007-0886-7) 18087688

[bib33] Li Y Huang J Yan Y Liang J Liang Q Lu Y Zhao L & Li H 2018 Preventative effects of resveratrol and estradiol on streptozotocin-induced diabetes in ovariectomized mice and the related mechanisms. PLoS One 13 e0204499. (10.1371/journal.pone.0204499) 30273360 PMC6166971

[bib34] Marchese E Rodeghier C Monson RS McCracken B Shi T Schrock W Martellotto J Oberholzer J & Danielson KK 2015 Enumerating β-cells in whole human islets: sex differences and associations with clinical outcomes after islet transplantation. Diabetes Care 38 e176–e177. (10.2337/dc15-0723) 26384388 PMC4613918

[bib35] Moede T Leibiger IB & Berggren PO 2020 Alpha cell regulation of beta cell function. Diabetologia 63 2064–2075. (10.1007/s00125-020-05196-3) 32894317 PMC7476996

[bib36] Mohan S Lafferty RA Flatt PR Moffett RC & Irwin N 2022 Ac3IV, a V1a and V1b receptor selective vasopressin analogue, protects against hydrocortisone-induced changes in pancreatic islet cell lineage. Peptides 152 170772. (10.1016/j.peptides.2022.170772) 35202749

[bib37] Patel SH O’Hara L Atanassova N Smith SE Curley MK Rebourcet D Darbey AL Gannon AL Sharpe RM & Smith LB 2017 Low-dose tamoxifen treatment in juvenile males has long-term adverse effects on the reproductive system: implications for inducible transgenics. Scientific Reports 7 8991. (10.1038/s41598-017-09016-4) 28827578 PMC5566418

[bib38] Plesner A Ten Holder JT & Verchere CB 2014 Islet remodeling in female mice with spontaneous autoimmune and streptozotocin-induced diabetes. PLoS One 9 e102843. (10.1371/journal.pone.0102843) 25101835 PMC4125302

[bib39] Richardson SS Reiches M Shattuck-Heidorn H LaBonte ML & Consoli T 2015 Opinion: focus on preclinical sex differences will not address women’s and men’s health disparities. Proceedings of the National Academy of Sciences of the United States of America 112 13419–13420. (10.1073/pnas.1516958112) 26534989 PMC4640753

[bib40] Saad MJ Folli F Kahn JA & Kahn CR 1993 Modulation of insulin receptor, insulin receptor substrate-1, and phosphatidylinositol 3-kinase in liver and muscle of dexamethasone-treated rats. Journal of Clinical Investigation 92 2065–2072. (10.1172/JCI116803) 7691892 PMC288376

[bib41] Sachs S Bastidas-Ponce A Tritschler S Bakhti M Böttcher A Sánchez-Garrido MA Tarquis-Medina M Kleinert M Fischer K Jall S, 2020 Targeted pharmacological therapy restores β-cell function for diabetes remission. Nature Metabolism 2 192–209. (10.1038/s42255-020-0171-3) 32694693

[bib42] Saeedi P Salpea P Karuranga S Petersohn I Malanda B Gregg EW Unwin N Wild SH & Williams R 2020 Mortality attributable to diabetes in 20–79 years old adults, 2019 estimates: results from the international diabetes federation diabetes atlas. Diabetes Research and Clinical Practice 162 108086. (10.1016/j.diabres.2020.108086) 32068099

[bib43] Saleh M Gittes GK & Prasadan K 2021 Alpha-to-beta cell trans-differentiation for treatment of diabetes. Biochemical Society Transactions 49 2539–2548. (10.1042/BST20210244) 34882233 PMC8786296

[bib44] Salpeter SR Walsh JM Ormiston TM Greyber E Buckley NS & Salpeter EE 2006 Meta‐analysis: effect of hormone‐replacement therapy on components of the metabolic syndrome in postmenopausal women. Diabetes, Obesity and Metabolism 8 538–554. (10.1111/j.1463-1326.2005.00545.x) 16918589

[bib45] Schwenk RW Baumeier C Finan B Kluth O Brauer C Joost HG DiMarchi RD Tschöp MH & Schürmann A 2014 GLP-1–oestrogen attenuates hyperphagia and protects from beta cell failure in diabetes-prone new Zealand obese (NZO) mice. Diabetologia 58 604–614. (10.1007/s00125-014-3478-3) 25527001 PMC4320309

[bib46] Sjögren LL Mørch LS & Løkkegaard E 2016 Hormone replacement therapy and the risk of endometrial cancer: a systematic review. Maturitas 91 25–35. (10.1016/j.maturitas.2016.05.013) 27451318

[bib47] Steinberg JR Turner BE Weeks BT Magnani CJ Wong BO Rodriguez F Yee LM & Cullen MR 2021 Analysis of female enrollment and participant sex by burden of disease in US clinical trials between 2000 and 2020. JAMA Network Open 4 e2113749. (10.1001/jamanetworkopen.2021.13749) 34143192 PMC8214160

[bib48] Stout MB Scalzo RL & Wellberg EA 2021 Persistent metabolic effects of tamoxifen: considerations for an experimental tool and clinical breast cancer treatment. Endocrinology 162 bqab126. (10.1210/endocr/bqab126) 34161568 PMC8282119

[bib49] Tanday N Flatt PR Irwin N & Moffett RC 2020a Liraglutide and sitagliptin counter beta-to alpha-cell transdifferentiation in diabetes. Journal of Endocrinology 245 53–64. (10.1530/JOE-19-0451) 31977315

[bib50] Tanday N Irwin N Moffett RC Flatt PR & O’Harte FPM 2020b Beneficial actions of a long-acting apelin analogue in diabetes are related to positive effects on islet cell turnover and transdifferentiation. Diabetes, Obesity and Metabolism 22 2468–2478. (10.1111/dom.14177) 32844576

[bib51] Therapontos C Erskine L Gardner ER Figg WD & Vargesson N 2009 Thalidomide induces limb defects by preventing angiogenic outgrowth during early limb formation. Proceedings of the National Academy of Sciences of the United States of America 106 8573–8578. (10.1073/pnas.0901505106) 19433787 PMC2688998

[bib52] Tiano JP & Mauvais-Jarvis F 2012 Importance of oestrogen receptors to preserve functional β-cell mass in diabetes. Nature Reviews. Endocrinology 8 342–351. (10.1038/nrendo.2011.242) 22330739

[bib53] Vargesson N 2015 Thalidomide‐induced teratogenesis: history and mechanisms. Birth Defects Research. Part C, Embryo Today: Reviews 105 140–156. (10.1002/bdrc.21096) 26043938 PMC4737249

[bib54] Vasu S Moffett RC Thorens B & Flatt PR 2014 Role of endogenous GLP-1 and GIP in beta cell compensatory responses to insulin resistance and cellular stress. PLoS One 9 e101005. (10.1371/journal.pone.0101005) 24967820 PMC4072716

[bib55] Xu B Allard C Alvarez-Mercado AI Fuselier T Kim JH Coons LA Hewitt SC Urano F Korach KS Levin ER, 2018 Estrogens promote misfolded proinsulin degradation to protect insulin production and delay diabetes. Cell Reports 24 181–196. (10.1016/j.celrep.2018.06.019) 29972779 PMC6092934

[bib56] Yokomizo H Inoguchi T Sonoda N Sakaki Y Maeda Y Inoue T Hirata E Takei R Ikeda N Fujii M, 2014 Maternal high-fat diet induces insulin resistance and deterioration of pancreatic β-cell function in adult offspring with sex differences in mice. American Journal of Physiology-Endocrinology and Metabolism 306 E1163–E1175. (10.1152/ajpendo.00688.2013) 24691028

[bib57] Zhao L Wang B Gomez NA de Avila JM Zhu MJ & Du M 2020 Even a low dose of tamoxifen profoundly induces adipose tissue browning in female mice. International Journal of Obesity 44 226–234. (10.1038/s41366-019-0330-3) 30705393 PMC6669124

[bib58] Zucker I & Prendergast BJ 2020 Sex differences in pharmacokinetics predict adverse drug reactions in women. Biology of Sex Differences 11 32. (10.1186/s13293-020-00308-5) 32503637 PMC7275616

